# "Clicks, likes, shares and comments" a systematic review of breast cancer screening discourse in social media

**DOI:** 10.1371/journal.pone.0231422

**Published:** 2020-04-15

**Authors:** Bence Döbrössy, Edmond Girasek, Anna Susánszky, Zsuzsa Koncz, Zsuzsa Győrffy, Virág Katalin Bognár

**Affiliations:** Institute of Behavioural Sciences, Semmelweis University, Faculty of Medicine, Budapest, Hungary; Medical University of Vienna, AUSTRIA

## Abstract

**Background:**

Unsatisfactory participation rate at population based organised breast cancer screening is a long standing problem. Social media, with 3.2 billion users in 2019, is potentially an important site of breast cancer related discourse. Determining whether these platforms might be used as channels by screening providers to reach under-screened women may have considerable public health significance.

**Objectives:**

By systematically reviewing original research studies on breast cancer related social media discourse, we had two aims: first, to assess the volume, participants and content of breast screening social media communication and second, to find out whether social media can be used by screening organisers as a channel of patient education.

**Methods:**

We followed the Preferred Reporting Items for Systematic Reviews and Meta-Analyses (PRISMA). After searching PubMed, ScienceDirect, Web of Science, Springer and Ebsco, 17 studies were found that met our criteria. A systematic narrative framework was used for data synthesis. Owing to the high degree of heterogeneity in social media channels, outcomes and measurement included in this study, a meta-analytic approach was not appropriate.

**Results:**

The volume of breast cancer related social media discourse is considerable. The majority of participants are lay individuals as opposed to healthcare professionals or advocacy groups. The lay misunderstandings surrounding the harms and benefits of mammography is well mirrored in the content of social media discourse. Although there is criticism, breast cancer screening sentiment on the social media ranges from the neutral to the positive. Social media is suitable for offering peer emotional support for potential participants.

**Conclusion:**

Dedicated breast screening websites operated by screening organisers would ensure much needed quality controlled information and also provide space for reliable question and answer forums, the sharing of personal experience and the provision of peer and professional support.

## Introduction

In 2018, there were more than 2 million new cases of breast cancer. Not only is it the cancer affecting women most frequently, it is also the second most common cancer overall.[1] Although recommended guidelines vary somewhat, breast cancer screening with mammography has the support of national and international cancer organisations and agencies to combat breast cancer mortality. Among others, the WHO, the International Agency for Research on Cancer (IARC), the European Commission Initiative on Breast Cancer all recommend participation in organised, population based screening programmes. As a consequence, in 2016, among the 67.5 million 50–69-year-old women living in the EU, 63.9 million (94.7%) were residents of Member States which had implemented or were piloting such programmes.[2] In all member states with the exception of Romania, screening eligible women (in most countries this means 50 to 69 year old women not treated for breast cancer, but in some countries it starts at 45 years and in others 74 year old women are also included) receive an invitation letter biennially (in the UK and Malta every 3 years) with a fixed but modifiable appointment or the instruction to get one, and participants do not have to pay for the screening mammography. [3]

There is evidence that invitation has increased participation rates and reduced inequalities in access.[4] [5]

As many of the articles reviewed concern the USA, it is worth briefly reviewing the status of breast cancer screening there. Although there is no centrally organised invitation based breast cancer screening programme in the USA, uninsured and poor women can get free screening through the National Breast and Cervical Cancer Early Detection Program. [6] The Affordable Care Act commends private insurance companies to make mammography screening freely available without co-payment in their packages.[7]

Despite the efforts, breast cancer screening participation is low in many countries, reaching 60%, 10 percentage points below the 70% participation rate deemed acceptable by the WHO. [8] It is also low among numerous socio-demographic groups, like members of ethnic minorities, rural residents and elderly women. [9]

Even with the support for it from national and international agencies, making informed breast cancer screening decisions on the individual level are not easy. On the one hand, there is the benefit of identifying breast cancer in its earliest and most treatable phase. On the other, there are factors such as overdiagnosis, overtreatment, radiation risk, the conflicting recommendations on what age to start screening, discomfort and anxiety associated with the examination that must be considered. Scientific studies critical of the safety and effectiveness of screening with mammography, most notably Miller et al (2014) [10] and Gøtzsche PC, Jørgensen KJ, (2013)[11] received thorough publicity in the popular media.

According to the literature on breast screening adherence, there are three determinants of a woman's commitment to cancer screening: socio-demographic factors, cognitive variables (beliefs and attitudes, risk perception, knowledge), and socioemotional variables (social relations, emotional dispositions, emotion regulation styles). [12]

Our hypothesis is that social media may potentially have a major influence on these variables by facilitating the forming of communities and sharing knowledge online, reaching large segments of the population.

The usage of Social media use is increasing globally. According to 2019 data, there are 3.2 billion social media users around the world., which is about 42% of the current population.[13]

Our review only identified original studies dealing with Facebook, Twitter, YouTube and QUORA (a question and answer website). In 2019, Facebook had over 2.4 billion users, worldwide as of October 2019, YouTube 2 billion, and Twitter had 3.3 million users.[14] Quora had 326 million registered users in 2018. [15]

Traditionally it was young adults who started using social media. They still make up the majority of users, but older adults are catching up as well. In the USA in 2019, 69% of the 50–64 year olds and 40% of the 65+ population used at least one type of social media.[16]

There is also evidence that socio-demographic groups less likely to attend breast cancer screening may be reached on social media. Pew Research Center data from 2015 shows that in the US 56% of those living in the lowest-income households use social media. There are no notable differences in social media use by ethnicity. 58% of rural residents use social media, which is only slightly less than urban social media use (64%). [17]

Social media opens a new dimension in healthcare to be used by all stakeholders to communicate and orientate about health issues. Smailhodzic and his colleagues found in their systematic review that social media use by patients could lead to an increased partnership in the healthcare professional—patient relationship through more equal communication between them. [18]

Increasing screening attendance is not only important on the level of the individual, but also on the system level as well. It is vital for the efficient functioning of the large and expensive population based breast cancer screening programs operated in many European countries. [19]

The aims of the present study are twofold. The first aim of this systematic review is to learn about the volume, participants, nature and content of breast screening related discussions on the social media.

The second goal of the present paper is to examine and synthetize the existing results in order to gather evidence on the feasibility of using social media as a channel through which potential participants can be reached and empowered through information and support to make the screening decision most beneficial to them.

## Methods

This systematic review followed the Preferred Reporting Items for Systematic Reviews and Meta-Analyses (PRISMA).[20] The PRISMA flow chart with the numbers can be found in the S1 Appendix.

### Eligibility criteria

To be included in this review, studies had to 1. be published in peer reviewed journal between 2009–2019, 2. be original studies, 3. be written in English, 4. examine breast cancer screening issues on Social Media platforms.

### Search strategy and study selection

The first step (screening stage 1) in this process was to search the following electronic databases: PubMed, ScienceDirect, Web Of Science, Springer and Ebsco. The search criteria used were: “breast cancer” AND “screening” AND”social media”;”mammography” AND “social media”; “Facebook/Twitter/Instagram/YouTube” AND “screening” AND “breast cancer”, alternatively AND “mammography”. This search yielded 116 citations. During the duplicate filtering 37 further research matches were excluded because they were found more than once.

**Fig 1:**
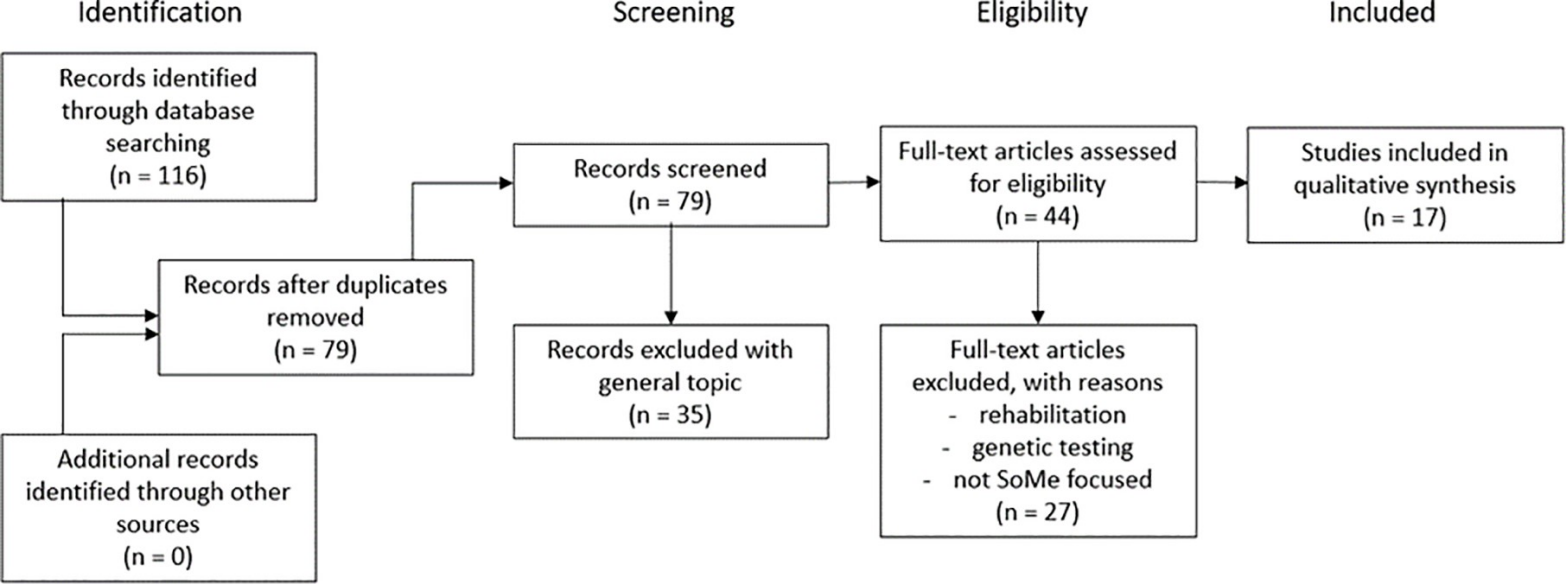
Prisma flowchart.

At screening stage 2, we made further exclusions. These secondary exclusion criteria were: papers focused on rehabilitation after diagnosed and treated breast cancer, papers about genetic screening, and papers, where social media use were not in the primary focus were omitted. At this stage, 17 papers remained to be examined for further analyses. The selection process can be seen on Fig 1.

Two researchers (BV and DB) independently screened each of the titles and abstracts from the initial literature search against the inclusion criteria. Authors GE and GYZS served as reviewers. Full text articles for the approved articles were than screened independently by 2 researchers. Discrepancies about inclusion were resolved by discussion, and a third reviewer was brought into the discussion if necessary.

### Data extraction

The data extracted included the studied site, research type, methods, sample, research objects/questions, type of social media channels, type of analyses performed and outcomes.

For the purpose of this review, social media was defined by the Merriam-Webster dictionary: “forms of electronic communication through which users create online communities to share information, ideas, personal messages, and other content”. Based above mentioned definition, we included the following platforms in the analyses:

- **Social networking sites: Facebook**
- **Micro-blogging sites–**Twitter
- **Video sharing sites–**YouTube,
- **Question and Answer website -**

### Data synthesis

A systematic narrative framework was used to synthesize the data. Owing to the high degree of heterogeneity in social media channels, outcomes and measurement included in this study, a meta-analytic approach was not appropriate. Following the systematic narrative framework for literature reviews, the results of included studies were synthesized and presented without reference to the statistical significance of the findings.

For a summary all the articles are reviewed, please refer to S1 Table.

## Results

From all the social media platforms, there were only studies on Twitter, YouTube, Facebook and the Quora question and answer website. 3 studies dealt with a dedicated breast cancer screening hub which was in the process of development in the UK. (Word Of Mouth Mammogram E-Network, a Web-based resource to support decision making regarding breast cancer screening.) Two papers researched social media in general and one used an experimental design with a simulated online forum.

### Volume of discourse

Information about the volume of discourse, the number of users potentially seeing posts and the number of times posts appear (impressions) is vital in determining whether messages may spread to sufficiently large segments of the target audience. This data is just as important as the number of posts per topic and the number of people sharing them, as many people only read posts, without reacting to them. Simply reading posts may also influence screening behaviour. For social media, the reach of a message is estimated by multiplying the number posts by the number of followers the users sharing it have (people who potentially will see these posts). This is the number of times messages appear to users. Not all the studies calculated reach and impression but report only the number of posts and the number of users posting them. The following table (S1 Table) presents data on the volume of breast cancer screening related discourse.

Thackeray, R., Burton, S. H., Giraud-Carrier, C., Rollins, S., & Draper, C. R. (2013) report that 797,827 unique users tweeted 1,351,823 breast cancer related tweets in a single breast cancer awareness month.[21] The reach of a message was estimated by multiplying the number of breast cancer related tweets by the number of followers the user has (who potentially will see the tweets). This number is a staggering 3,028,451,603. This is the number of times all the breast cancer messages appeared to users. The study of Huesch, M., Chetlen, A., Segel, J., & Schetter, S. (2017) provide evidence of 1.7 million unique breast cancer screening related interactions on Facebook by more than 1.1 million female Facebook users with mammography mentioned in 16% between November 15 and December 15, 2016.[22] 173 mammography related YouTube videos made by lay people and health professionals had 23,166,120 views up to 2015.[23]

### Participants in the discourse

**Fig 2:**
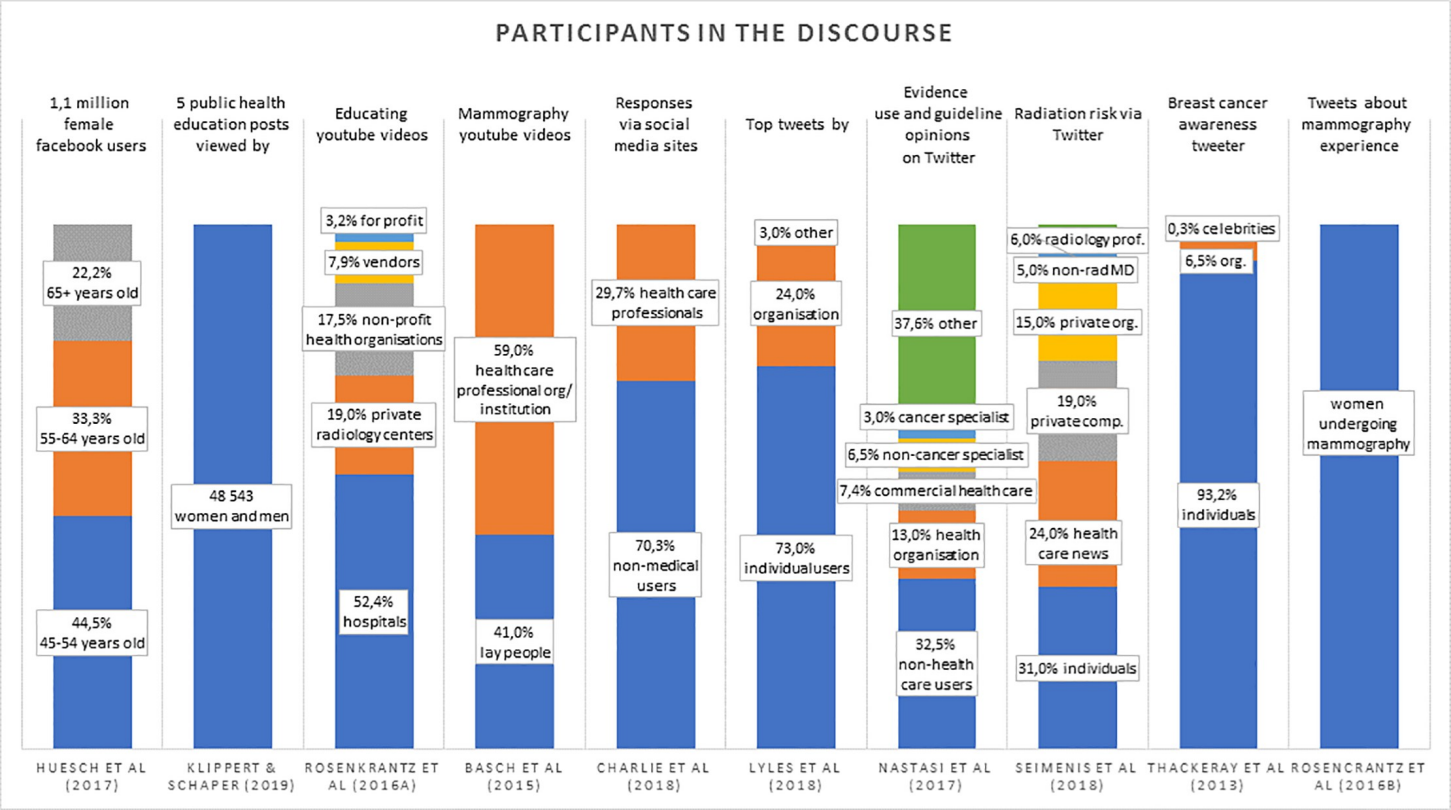
(Participants in the discourse) should be linked here.

The majority of participants in breast cancer related discourse are non-healthcare professional lay individuals as opposed to healthcare organisations, healthcare professionals or breast screening advocacy groups. Indeed, many of the reviewed articles mention the scarcity of professional input in the online breast screening social media discussions and call on professionals knowledgeable about screening to be more active (S3 Table) (Fig 2).

YouTube is the only platform where videos created by healthcare organisations (102 videos-59% of the total) outnumbered those made by lay people (71 videos-41% of the total). [23]On Twitter, although celebrities make up only a small part of the discourse participants, due to their vast number of followers, their posts and shares are potentially seen most often.

The three UK studies on lay and professional expectations about online breast cancer screening hubs specifically posed the question about professional online contributions and found that lay people as well as screening professionals feel the need for professionals to be more active to safeguard the quality of information online[24–26].

### The content of discourse

For clarity, the presentation of results on the nature of the online breast screening discourse is organised by the information content and its validity, mammogram related sentiment and content of sharing of the participants’ own screening related experiences

#### a. The scientific validity of breast cancer screening related information shared on social media

The results of the reviewed studies indicate that there is lay confusion surrounding breast cancer screening in social media discourse. As stated by Nastasi, A., Bryant, T., Canner, J. K., et al. (2017) in their own Twitter study, *“Non-healthcare users comprise a significant proportion of participants in mammography conversations, with tweets often containing claims that are false, not explicitly backed by scientific evidence, and in favor of alternative “natural” breast cancer prevention and treatment.”[27]*.

The authors furthermore note that unscientific statements shared by lay people are frequently not minor misunderstandings but fundamental to the scientific rationale of screening. The three small-scale UK studies that asked potential users and screening providers about online breast cancer discourse reported that their respondents felt that the information pool online is confusing. Lay people and screening organisers voiced the opinion that there is much factually false and hence misleading information spread on social media. [24–26]

The online presence of scientifically unfounded and misleading breast cancer screening information (mammography causes breast cancer, breast cancer can be prevented by organic food) is evidenced by other studies, too. [27] Nastasi, A., Bryant, T., Canner, J. K., Dredze, M., Camp, M. S., Nagarajan, N. (2018) found that only 61% of the 323 tweets posted by lay people had scientific support. The scientifically false tweets are liked and shared as often as the reliable ones in this study. It came as no surprise that medical doctors had 1.87 times higher odds (95% CI, 0.69–5.07) of linking references to support their views and 11.70 times higher odds (95% CI, 3.41–40.13) of posting scientifically valid tweets than lay people.

The research of Seimenis, I., Chouchos, K., & Prassopoulos, P. (2018) on radiation related tweets found that 23% of the 427 tweets in totalare misleading and scientifically invalid.[28]

On Facebook 6% of all top interactions in the 35–54 age group contained links to a strongly anti-screening natural health advocacy website voicing views contrary to what is accepted by the scientific community [22] (S4 Table).

#### b. Main topics discussed

Charlie, A. M., Gao, Y., & Heler, S. L. (2018) [29] studied mammography related questions on QUORA and found that the most frequent topics of enquiry concentrated on mammographic efficacy (16 of 51 [31.4%]; 71,020 views), screening guidelines and procedures (10 of 51 [19.6%]; 51,108 views), and what may be expected at a mammography test. (9 of 51 [17.6%]; 18,695 views (S5 Table)

4 of 22 (18.2%) nonmedical professional respondents were overtly opposing mammography screening (eg, mammography is “unscientific and harmful,” causes “cancer,” “doesn’t reduce mortality,” and has “high false-positive rates”). 3 out of the four critical non-medical professionals referred to scientific studies published in journals to support their views.

Overall, the topic causing the biggest online debate was the changing of screening guidelines in the USA. The raising of screening age from 40 to 45 by the American Cancer Society (ACS) and from 40 to 50 by the United States Protective Services Task Force (USPSTF) has very little online lay or professional support. Squiers LB et al. (2011) looked specifically at this issue and established that out of the 82 tweets found 48.8% were neutral, 36.6% unsupportive of the changed guideline and just 2.4% supportive, while 12.2% were confused. [30] Regarding the 71 blog posts they reviewed, 17% say postponing the recommended age of screening leads to breast cancer deaths. 31% of the blogs argue that guideline changes are due to government rationing of resources. Only 14% of blog postings held that changes had a scientific rationale. 66.2% of blog post are negative. The guideline changes were very frequently discussed on Twitter and QUORA and have minimal support.

An advantage of social media is that one can easily link in articles and other material to support the stand taken on any given issue. This is common in the breast screening discourse, too. Health society websites, health society guidelines and journal articles were the most commonly used linked references for medical and nonmedical users both. Depending on the topic, 5%-20% of links are to peer reviewed journals.[27] Posts critical to screening often referred to journal articles, too, illustrating that there is scientific evidence for both for and against screening. As expected, this study also found that lay people are less likely to cite evidence in the form of links than are healthcare professionals.

Two studies looked at the content of mammography related videos on YouTube.

Basch, C. H., Hillyer, G. C., MacDonald, Z. L., Reeves, R., & Basch, C. E. (2015) analysed 173 mammography related videos on YouTube. They report that 93.6% of the videos contained general mammography information (this is the term used by the authors who did not specify what is meant by it). Only 35.3% discussed the issue of pain and 32.4% addressed issues of anxiety and fear was addressed by 29.5%. 46.2%. of the videos presented information about the test results. This means that many of the videos didn’t discuss important issues. The authors themselves note that there is ground for improvement in content of the videos. [23]

Rosenkrantz A B, Eugene Won E, Doshi AM (2016) looked only at mammography related videos produced by health professionals and healthcare organisations and found important topics that were not covered by many educational videos. Dense breasts were not mentioned in any of the 11 mammography videos. Only 7 videos mentioned the possible need for further examination. Here again, some videos had important information missing and the authors themselves felt the need to call for improvements.[31]

Many of the tweets posted on the topic are not directly related to mammography. This is especially true for lay contributions in breast cancer awareness months. Wearing pink was the most frequent topic among lay people in Thackeray R, Burton SH, Giraud-Carrier C, Rollins S Draper CR (2013). Organisations tweeted more about fundraisers, early detection and diagnosis. [21]

The one study that analysed mammography related Facebook top post (33, 600 unique interactions) found that 36% of the interactions had to do with e-commerce activity trying to sell some product, 26% reshared celebrity breast cancer information and 15% were resharing of advocacy groups (mostly the American Cancer Society). [22]

#### c. Content regarding breast cancer screening sentiment

By breast cancer related sentiment what we mean is whether people regard breast screening mammography favourably or unfavourably. Negative sentiment may act as a deterrent to screening.

Looking at the cumulative results, we may say that in general, breast cancer screening sentiment expressed on social media ranges from neutral to favourable. Negative sentiments are expressed to a smaller degree. [27, 30] Although a minority of lay people voiced negative sentiments regarding the ineffectiveness and dangers of breast screening and content mentioning pain and anxiety associated with mammography screening as well as widespread criticism of US screening guidelines, screening is still generally viewed as beneficial.

The single Twitter sentiment analysis found there were 29,034 neutral, 21,561 positive and 4,069 negative tweets of the 61,524 breast screening related tweets from 17, September 2014 to 10, May 2015. [32]

Rosenkrantz AB, Eugene Won E, Doshi AM (2016) reported 15 times as many likes for mammography videos than dislikes. [31]

Seimenis I., Konstantinos Chouchos K., Panos Prassopoulos P (2018) identified 427 tweets related to the potential radiation risk of x-ray mammography and found that over the 3 year study period 42% of the tweets were favourable to mammography, 32% neutral and 26% were negative. It is interesting to note that of the favourable posts, 27% were from private companies, 22% from unspecified source, 18% from organisations, 17% from lay people, 8% from radiologists, 7% from physicians, and 1% from physicists.[28] For unfavourable posts, the ratio is completely different. Here, 62% of the posts are from persons, 20% are unspecified, 6% are from organisations, 6% are from private companies, 5% from physicians. (Percentages may not add up to 100% because of authors’ rounding in the original article) No negative posts came from radiologists. It is important to note that 26 of the roughly 100 negative posts were about breast thermography, which is not a scientifically recognised alternative of mammography in the early detection of cancer.

The term “digital” was mentioned in only 15% of the posts. The use of digital mammography is now the norm in all organised breast screening programmes as in this technology radiation exposure is significantly lower than in film mammography.

In their experiment of a simulated online breast screening related forum, Kimmerle J, Bientzle M and Cress U (2017) found that pro-mammogram arguments were seen as more relevant.[33] The stronger the screening intention, the more the participants recommended scientifically worded responses. Scientifically phrased inquiries get stronger recommendations from respondents participating in this experiment (S6 Table).

### Content about screening experience

A number of papers dealt with the subject of what women undergoing or having undergone mammography share about their own screening experience on social media. One study looking at what participants tweeted about their own mammography experience reviewed 464 relavent tweets. It found that the most common themes in the tweets were: the experience of breast compression (24.4%), giving advice to others to undergo screening (23.9%), testifying about the importance of the examination (18.8%), discussing the length of waiting times (10.1%), relief caused by negative results (9.7%), comments that the examination was not that bad (9.1%), examination anxiety (8.2%), interactions with staff (8.0%), the gown (5.0%), examination costs or access (3.4%), offering support to other participants or reaching out for it online (3.2%) screening denoting that one is getting old (2.4%), and the description of the waiting room. (1.3%).[34]

Humour was used in 31.9% of the tweets, 56.1% of which was related to compression. Tweets of first time mammography participants wrote more about compression when compared to those who had had the examination before (16.4% vs 9.1%, respectively) and that the test was not that bad (26.2% vs 7.6%).

As approximately 15% of tweets mentioned the radiology clinic where the test was performed by name, Twitter can be a source of good or bad publicity.

Lyles, C. R., López, A., Pasick, R., & Sarkar, U. (2013) found that 68 (25%) breast cancer screening top tweets are on personal experiences.[35] 7 tweets expressed a specific negative feeling, mostly about pain and discomfort, towards the procedure and 6 tweets expressing fear and anxiety regarding the test results or breast cancer. Even when talking about fear and anxiety, some of these tweets also had a positive message ‘the mammogram was brutal! But so necessary’. 9 of the personal experience tweets discussed the results (all negative results and all supportive of screening.) 15 tweets were written to support or ask for support family or friends undergoing screening.

Nastasi, A., Bryant, T., Canner, J. K., et al. (2017) studied evidence use and guideline opinions on Twitter and found that among personal tweets (n = 167), 85 (50.9%) were about mammography appointments and 26 (15.6%) were about mammography results.[27]

Three small scale, non-representative UK studies looked at lay and professional attitudes regarding expectations towards a website dedicated to breast screening operated by screening providers with embedded social media functions that could support decision making regarding breast cancer screening.

The main outcome was that potential service users and health professionals felt the need for women to engage with other women in order to access experiential information, and with practitioners to access professional, factual information. These indicate that it is not only quality controlled professional information that screening providers and users expect from such a site but also to provide space for peer communication about their own screening experiences and the provision of emotional peer support as well. [25, 26]

One of the studies found that although women would like to read about the experiences of others, they are hesitant to share their own. [24] Providing anonymity is important to encourage women to share their experiences.

The importance of providing anonymity is supported by Charlie, A. M., Gao, Y., & Heller, S. L. (2018.) in their study of breast screening related questions and answers on QUORA.[29] Out of the 51 questions posted, 7 asked for help in report interpretation and about abnormal results. These ‘personal’ questions were all posted anonymously, while the other, more general questions about safety, guidelines, efficacy and costs were posted with names.

## Discussion

In principle, social media is an ideal channel to promote breast screening It is widely used, there are screening participants to share accounts of their screening experience and provide emotional peer support. Professionals can get involved in the discourse, too, with valid information. As research shows, social media is a major site for people seeking health related information and support. [36, 37]

There is also evidence that women from socio-demographic groups characterised by lower screening attendance are frequent on social media. [9]

Deshpande, M. and Look, K.A. (2017) found that members of ethnic minorities are active in sharing health related information on social media. A significantly higher percentage of Hispanics (17.8%) and other races (27.0%) chose to share health information on social media compared to African Americans (8.6%) and Whites (12.9%).[38]

After reviewing the 17 relevant original studies identified, we can conclude that the volume of breast cancer related social media is big enough to make it a good channel for reaching the target population.

The volume of breast cancer screening discourse indicates that that the topic generates intense interest.

As much of the information can be presented in pictures, animations and videos, people with limited literacy skills can also benefit from this format as opposed to purely written messages.

A benefit of social media is the possibility of linking materials. Besides blogs, and general news articles, 5%-20% of links are to peer reviewed journals.[27] This means that academic debate on the pros and cons of breast cancer screening may have a wider impact by reaching the general population as social media links. As the academic opinion on breast cancer screening is not univocal, scientific articles can be found as links on social media in arguments for and against breast cancer screening.[28]

Breast cancer screening social media communication is often confusing and unreliable, so professional involvement is needed. There are a few medical and screening professionals active on social media, but they are in the minority. Their presence and activity would be greatly needed to contribute quality information to help disperse the questionable content spread online.

Having access to a large volume of breast cancer screening related social media discourse in itself will not help women make informed screening choices, only quality controlled, scientifically valid information will have that effect. Social media provides a forum where anyone can post more or less anything they wish without the need of verification. In the results section, we have provided evidence that scientifically invalid statements are liked and shared as much as valid information suggesting that many people can’t differentiate between what they may trust and what they shouldn’t. The only way this may potentially be combated is through the ‘policing’ of the online content by screening knowledgeable health professionals. Not only could they upload scientifically correct posts, they could also comment and refute misleading and false messages.

The present study reveals that breast cancer screening social media use by lay people may not only provide beneficial effects: the misinformation, uncertainty and lack of awareness of clear guidelines may act as a deterrent to screening participation, and lead to an immense challenge to the medical workforce if they are to help potential participants make fact based informed screening decisions. [22, 23, 27]

The majority of participants in breast cancer related online discourse are lay people. They are responsible for most of the shared misinformation.

Celebrities taking part in the discourse are very influential as posts shared by them reach many people due to their large following. The so called “Angelina Jolie effect is a case in point.

On the 14^th^ of May, 2013, this A-list Hollywood actress and activist informed the world through a long New York times article that she had undergone an elective double preventive mastectomy to eliminate the 87% probability of breast cancer due to the BRC1 faulty gene. [39] On the day the article was published, the preventive mastectomy site of the American National Cancer Institute received 69225 visits, 795 times more than a week before.[40]

There is research evidence that the hype the case of this celebrity caused in the mass and social media lead to an increase in the demand for BRCA tests as well as the number of preventive mastectomies themselves in England [41], in New York and in New South Wales. [42]

One barrier to screening participation is socio-emotional variables. There is research evidence that women who lack social support are less likely to attend.[12]

One important function of social media is the possibility of forming supporting peer networks. Users are able to create a community where they get the chance to share experiences and support. This may be very effective to provide the social, emotional care needed to make screening decisions. Already in 2012 there were over 8 million participants combined in Twitter and Facebook breast cancer groups, offering each other experiential information and emotional support.[35, 43]

It must be remembered nevertheless that breast screening participation only happens to women every two or three years, depending on the screening guidelines in the country. Being a screening participant is a transient status, unlike being a breast cancer survivor, so the formation of online communities is much less likely. This doesn’t mean that peer support is not offered. There is evidence from the studies reviewed of peer social support offered on social media encouraging others to participate.[34]

One potentially good solution would be the use of dedicated breast screening hubs or Digital Support Networks by screening organisers. Not only would these hubs have quality controlled information, they would also provide the opportunity for reliable question and answer forums, the sharing of personal experience and the provision of peer and professional support. With super users sharing their own experiences and responding to others, the online peer conversation may be kept going.

Building partnerships with celebrities is also important for social media public health outreach. As celebrities have many followers, whatever they share will have a big impression as it will be seen by many people.

A final lesson learnt is that social media is a good source of research information on the screening related beliefs, attitudes and literacy of the target population. There is a big body of breast cancer related discourse online, ready to be studied and utilised without having to conduct surveys and interviews.

In conclusion we may state that there is evidence presented in our review that social media is a channel which public health authorities and breast cancer screening organizers could use to reach out and aid underscreened people by providing solid information in an easy to understand way to help them make the screening decision which is the best for them. Social media is also suitable for offering peer emotional support for potential participants. This is not only a possibility it is also a necessity as the online confusion relating to the harm and efficiency of mammography screening may potentially deter women from attending screening.

A major weakness of organised breast cancer screening programmes is low attendance rates which is partly due to ineffective communication strategies. The review provided evidence of the importance of social media in public health efforts.

Social media has already been applied as a setting in health promotion. Although it offers many opportunities, social media alone cannot implement behavioural modification and advance health outcomes; rather, it can be a great instrument to reach out to the online community and communicate about health programs and services. As social media use varies by age, education and socioeconomic status, breast cancer screening social media communication should be tailored to the target population. [44]

### Strengths and limitations

To our knowledge, this is the first ever systematic review of research dealing with breast cancer screening related discourse in the social media.

The 17 original research studies reviewed here vary greatly in the type of social media examined, in research methodology in content researched and in research questions asked. Hence, synthesizing the data was not feasible and even general comparison was difficult. This made the systematic presentation of the results somewhat cumbersome.

Some of the studies were not exclusively about breast cancer screening in the social media but were still included in the review because information on this topic could be isolated from it. Many of the studies were exploratory in nature, collecting information on top posts only (the ones most likely to reach the most people), so the analysis is not descriptive of the given social media per se.

4 of the 5 focus group and survey studies used very small, non-representative samples, so the results are indicative at best. (Robinson, L., Griffiths, M., Wray, J., Ure, C., Shires, G., Stein-Hodgins, J. R., … & Hilton, B. 2015), on what potential users and health professionals would expect from a breast screening dedicated webpage (Galpin, A., Meredith, J., Ure, C., & Robinson, L. 2017) Kimmerle, J., Bientzle, M., & Cress, U. (2017) on the wording of posts. (Klippert, H., & Schaper, A. (2019) had only 49 returned questionnaires to evaluate their Facebook campaign study.

## Supporting information

S1 Appendix PRISMA 2009 checklist. (DOC)

S1 Table Evidence table. (DOCX)

S2 Table The volume of discourse. (DOCX)

S3 Table Participants in the discourse. (DOCX)

S4 Table Scientific validity of discourse. (DOCX)

S5 Table Main topics discussed. (DOCX)

S6 Table Screening sentiment. (DOCX)
