# Supplementary material for: "Clicks, likes, shares and comments" a systematic review of breast cancer screening discourse in social media
Source: PLoS One. 2020 Apr 15;15(4):e0231422. doi: 10.1371/journal.pone.0231422 (PMC7159232; doi:10.1371/journal.pone.0231422)
Supplement: S1 Table — (DOCX) [file pone.0231422.s002.docx]

*1. table Evidence table*

| Title | Research question | Social media type | Methodology  And strength of evidence | Volume of discourse | Participants in discourse | Content of discourse |
| --- | --- | --- | --- | --- | --- | --- |
| Huesch, M., Chetlen, A., Segel, J., & Schetter, S. (2017). Frequencies of private mentions and sharing of mammography and breast cancer terms on Facebook: a pilot study. *Journal of medical Internet research, 19*(6), e201. | What are the most common BCS terms and phrases used on Facebook and the most commonly shared website links by sharer characteristics? | Facebook | Content analysis of BCS related interactions and stories on Facebook between November 15 and December 15, 2016.  Strong evidence | 1.7 million unique interactions  by more than 1.1 million female Facebook users.  There were 59,000 unique stories  On average 1.5 times shared,  8 times commented, and reacted to more than 20 times | 1.1 million female Facebook users.  Many of the women were screening aged:  0.4M 45-54 years old  0.3M 55-64 years and 0.2M by 65+ | Mammography was mentioned in 16% of the interactions.  links shared in top interactions: 36% e-commerce,  26 % celebrity breast cancer information,  15% advocacy groups.  6% of all top interactions in the 35-54 age group is strongly anti screening |
| Klippert, H., & Schaper, A. (2019). Using Facebook to communicate mammography messages to rural audiences. *Public Health Nursing*, *36*(2), 164-171. | What is the highest level of engagement achieved by a mammography campaign on Facebook? | A paid advertisement tailored mammogram campaign boosted on Facebook | 5 posts and a questionnaire boosted towards 40+ men and women in 9 counties of Idaho.  An online questionnaire was administered on intent to get screened.  Strong evidence | There were 48,503 views of posts and  36,583 women were reached. There were  1,189 reactions, mostly likes., Posts were shared 204 times.  Post received 43 comments,  49 questionnaires were returned. | An Idaho public health department sent the posts to  40+  Hard to reach, under screened rural women  and men | Reaction:1,189 ( likes mostly)  There were 43 comments:  4 about having booked a mammogram appointment.  82% intends screening.  43% wants more mammography information on Facebook. 45% were neutral about the topic. |
| Rosenkrantz, A. B., Won, E., & Doshi, A. M. (2016). Assessing the content of YouTube videos in educating patients regarding common imaging examinations. *Journal of the American College of Radiology*, *13*(12), 1509-1513. | What is the content of YouTube videos created by healthcare organisations about common imaging, including mammography? | YouTube videos created by healthcare organisations on common imaging. | Content analysis of official imaging videos on YouTube.  The videos were characterised by source, length, views, likes, dislikes, comments  Strong evidence | 11 videos made were made by healthcare organisations. There were 151,664 views,  86 likes, 22 dislikes  and 10 comments | The video sources for all imaging video were:  52.4% hospitals,  19% private radiology centers  17.5% other nonprofit health organizations  7.9% vendors 3.2% other for-profit health organizations | Unrobing, avoiding deodorant, and possible additional images were all mentioned by 63.6% of the mammography videos; but dense breasts were mentioned by none of them  . |
| Basch, C. H., Hillyer, G. C., MacDonald, Z. L., Reeves, R., & Basch, C. E. (2015). Characteristics of YouTube™ videos related to mammography. *Journal of Cancer Education*, *30*(4), 699-703. | Analyse coverage of mammography screening in popular YouTube videos made by professionals and consumers | YouTube | Content analysis and characterisation of videos  Strong evidence | The 173 videos received 23,166,120 views.  The mean number of views of the videos was 123,341, about 4500 views per month | 59% of the videos were authored by healthcare professionals 41% were created by lay people | 93.6 % had general information.  46.2% presented information about the test results  25% covered medical or family history  35.3 % discussed pain  32.4 % anxiety  29.5 %. Fear 46.2 %. Results.  Comments were on safety, pain, and radiation.  . |
| Charlie, A. M., Gao, Y., & Heller, S. L. (2018). What do patients want to know? Questions and concerns regarding mammography expressed through social media. *Journal of the American College of Radiology*, *15*(10), 1478-1486. | What are the Breast Cancer Screeningrelated questions and concerns users have on Quora? | Quora question and answer website | Content analysis of posted questions (June 2010 to February 2017)  Compared respondent type, screening recommendations and evidence.  Strong evidence | There were 51 questions and 172 responses to them and  197,620 views overall. | All 51 questions were from lay people.  In all, there were 172 responses to them.  51 responses from 19 physicians and 6 registered nurses,  121 from non medical professional users. | Mammographic efficacy (16 of 51 [31.4%]) and screening guidelines (10 of 51 [19.6%]) were the most common topics.  7 questions asked for help in result interpretation, 9 enquired about what to expect at screening, 4 about safety and 2 asked for advice  most professionals did not agree with the guidelines not recommending screening to start at age 40.  Among lay participants, 4 of 22 (18.2%) were against screening mammography |
| Galpin, A., Meredith, J., Ure, C., & Robinson, L. (2017). “Thanks for letting us all share your mammogram experience virtually”: developing a web-based hub for breast cancer screening. *JMIR cancer*, *3*(2), e17. | What do potential users want from a Web-based resource for breast cancer screening? | Designing  Word Of Mouth Mammogram E-Network,  a Web-based resource to support decision making regarding breast cancer screening | Content analysis of discussion on a closed Facebook user design group to find out about requirements, anxieties etc. of potential users and Healthcare professionals.  23 women answered a questionnaire  Weak evidence | At the end of the research, the user design Facebook group had 71 health professionals and potential service users | Convenience sample of women and health professionals | Potential service users and health professionals felt the need for women to engage with other women in order to access experiential information, and with practitioners to access professional, factual information.  Both agreed that there is a lot of misinformation online. |
| Robinson, L., Griffiths, M., Wray, J., Ure, C., Shires, G., Stein-Hodgins, J. R., ... & Hilton, B. (2015). Preparing women for breast screening mammography: a feasibility study to determine the potential value of an on-line social network and information hub. *Radiography*, *21*(4), 308-314. | What should the hub encompass according to women; how would they network with other women how should information be presented, should health professionals be present? | a dedicated breast screening hub or Digital Support Network (DSN) | 94 survey questionnaires and two focus groups based on age group  Weak evidence | 10 women in two age based focus groups (18-44, 45-84)  94 questionnaire respondents. | Convenience sample of women | Women require factual information and having health professionals involved in the DSN. User generated experiences are important, but most respondents want to read about it, not share their own.  Having super-users are important .  Challenge is privacy, |
| Scragg, B., Shaikh, S., Shires, G., Hodgins, J. S., Mercer, C., Robinson, L., & Wray, J. (2017). An exploration of mammographers' attitudes towards the use of social media for providing breast screening information to clients. *Radiography*, *23*(3), 249-255. | How does the NHS breast screening workforce feel about engaging potential participants online? | Social media in general | 78 breast cancer screening professionals were asked about challenges they face in using social media + | 78 breast cancer screening professionals from different UK regions and with different roles. | Invited screening professionals in various roles. | The idea was liked but there are challenges:  Privacy, difficulty with online communication, time lack of infrastructure |
| Deshpande, M., & Look, K. A. (2017). Relationship between health information sharing behavior using social media and breast cancer Screening. *INNOVATIONS in pharmacy*, *8*(2). | What is the Social media health information sharing behavior of the breast screening eligible population? Is sharing health information on social media associated with BCS participation? | Social media in general | Data from the 2013 Health Information National Trends Survey using descriptive statistics and bivariate logistic regression to evaluate the association between sharing health information on social media and receipt of mammogram  strong evidence | 13% of breast cancer screening aged women used social media to share health information.  Hispanics and other races were more likely to share than Whites or Afro-Americans | women aged 50- 74 with no history of breast cancer. (n-731) | The analysis found no association between breast cancer screening attendance and health information sharing behaviour.  . |
| Kimmerle, J., Bientzle, M., & Cress, U. (2017). “Scientific evidence is very important for me”: The impact of behavioral intention and the wording of user inquiries on replies and recommendations in a health-related online forum. *Computers in Human Behavior*, *73*, 320-327. | How do participants communicate about simulated mammography screening In an experimental setting on a simulated online forum? | Experimental online forum  Not real participants, not real forum | Experiment. 61 women engaged in a simulated online forum were presented with and contra arguments about screening. The researchers manipulated scientific vs holistic arguments in the comments.  Weak evidence | 61 women in an experimental setting | Women between the ages of 18-46 responded to pro screening and anti screening holistic or scientifically worded pre- written posts. | Pro-mammography arguments were seen as more relevant by respondents. The stronger the screening intention, the more the participants recommended scientifically worded responses. Scientifically phrased inquiries get stronger recommendations. |
| Wong, K. O., Davis, F. G., Zaïane, O. R., Yasui, Y., Dietz, J., Aveiro, D., ... & Bernardino, J. (2016, November). Sentiment analysis of breast cancer screening in the United States using Twitter. *KDIR* (pp. 265-274). | How is screening sentiment expressed on twitter?  Is expressed sentiment related to screening uptake? | Twitter | A description of the temporal, geospatial, and thematic patterns of public breast cancer sentiment of tweets in the U.S. Used CDC data to get breast screening uptake rates on the state level.  Weak evidence | From 17, September 2014 to 10 ,May 2015 researchers found 61,524 Beast Cancer Screening tweets | NA | There were 29,034 neutral, 21,561 positive and 4,069 negative tweets.  There was a significant positive association between neutral sentiment and clinical breast examination uptake. Average negative sentiment scores negatively associated with mammogram and clinical breast examination uptakes.  No positive association was found between positive sentiment and mammography and CBE |
| Squiers, L. B., Holden, D. J., Dolina, S. E., Kim, A. E., Bann, C. M., & Renaud, J. M. (2011). The public's response to the US Preventive Services Task Force's 2009 recommendations on mammography screening. *American journal of preventive medicine*, *40*(5), 497-504. | What is the volume and framing of the public mammography discourse on recommendations? | Twitter, blogs, news media  (the data on news media is not analysed here.) | 1.Content analysis of newspaper articles, blog posts, and tweets around the announcement of new guidelines  OK evidence | 82 tweets and 71 blog posts discussing screening recommendations in the period of November 2009 to January 2010 | NA | 48.8% of the tweets were neutral, 36.6% unsupportive of the changed guideline and just 2.4% supportive, while 12.2% were confused  Blogs: 17% say postponing the recommended age of screening leads to breast cancer deaths. 31% say changes are due to government rationing of resources. 14% changes had a scientific rationale.  66.2% of blog post are negative |
| Lyles, C. R., López, A., Pasick, R., & Sarkar, U. (2013). “5 mins of uncomfyness is better than dealing with cancer 4 a lifetime”: an exploratory qualitative analysis of cervical and breast cancer screening dialogue on Twitter. *Journal of Cancer Education*, *28*(1), 127-133. | Is there cancer screening content on twitter? Is there enough of it for analysis? What is the nature of this content? | Twitter | The study is an exploratory qualitative content analysis of tweeter messages relating to Pap smear and mammography Strong evidence | There were 271 mammography related tweets in 5 weeks in 2012. | 73% of top tweets came from individual users, 24% from organisations and 2% from news sources. | 25 % of top tweets are on personal experiences, there are some negative tweets about procedure.  promotional tweets are 18 % of the total.  Guideline critical tweets are (9),  Jokes and sexual content is also present. |
| Nastasi, A., Bryant, T., Canner, J. K., Dredze, M., Camp, M. S., & Nagarajan, N. (2018). Breast cancer screening and social media: a content analysis of evidence use and guideline opinions on Twitter. *Journal of Cancer Education, 33*(3), 695-702. | How do tweeters use evidence in twitter discourse and what do they write about the changed USA cancer screening guidelines? | Twitter | The time frame was 5 November 2015 to 11 December 2015. Prospective mixed-method content tweet analysis of data related to user type, content, evidence use, and guideline opinions  Strong evidence | There were 1345 mammography related tweets authored by 995 unique users in the study period. | Non-healthcare users made up 32.5% of the total,  health organizations 13.0%;  non-cancer specialist 6.5%,  cancer specialists 3% | 71.3% of the tweets had a link,  24.8% to general news sites,  21.5% to healthcare websites,  5.2% of tweets to journal articles or guidelines.  Testable claims tweets were 37.9% of all, BC news 12.9%.  45.3% of tweets are on BC research. 81.6% of claims were sound scientifically 14.7% non  Physicians posted scientifically valid tweets (OR 11. 7)  Scientifically valid and faulty posts were just as likely to be retweeted.  Only 61% claims related non-healthcare tweets had scientific support. |
| Seimenis, I., Chouchos, K., & Prassopoulos, P. (2018). Radiation risk associated with X-Ray mammography screening: communication and exchange of information via Tweets. *Journal of the American College of Radiology*, *15*(7), 1033-1039. | What are the informational and opinion trends on Twitter on radiation harm in screening mammography? | Twitter | Content analysis of tweets containing keywords ‘mammography ‘and ‘radiation’ by user type, content, context, linked source  (2014-2016)  Strong evidence | 427 tweets were found on the topic of mammography radiation from 329 unique users | 31% of all top tweets were by individuals. 24% were healthcare and news feeds. Private companies and organizations had 19% and 15%. Specialised radiology professionals were responsible for 5% of tweets. Non radiologist MDs posted 7% of tweets. | 25% of tweets were remarks with no linked sources  20% of the linked sources were peer reviewed journals, 23% were no- peer reviewed, 10% each were blogs, advertisements and informative websites.  42% of the tweets were favourable to mammography, 32% neutral and 26% negative.  62% of unfavourable post were from the general public while only 17% of the favourable posts were by them.  . 73% of total tweets were informative,  23% were misleading and 4% were questions |
| Thackeray, R., Burton, S. H., Giraud-Carrier, C., Rollins, S., & Draper, C. R. (2013). Using Twitter for breast cancer prevention: an analysis of breast cancer awareness month. *BMC Cancer, 13*(1), 508. | What is the frequency of breast cancer tweets during Breast Cancer Awareness  Month? Are individuals, organizations, or celebrities more likely to tweet? What is the reach and content? | Twitter during BCAM | This was a cross-sectional, descriptive study. It collected and categorised breast cancer- related tweets from 26 September - 12 November 2012  Strong evidence | 797,827 unique users  tweeted 1,351,823 breast cancer related tweets.  The messages could potentially have been seen 3,028,451,603 times.  Impressions | 93.2% of the tweeters were individuals,  6.5 % organizations, and 0.3% by celebrities. Organizations tweeted 10.7% of all the tweets. Celebrities 0.4%, Individuals 88.9%, | Wearing pink, promoting breast cancer awareness walks and fundraisers were the most usual tweet topics.  Organisations and celebrities were mostly tweeting about fundraisers, early detection, and diagnoses. Individuals were tweeting about clothing and walks |
| Rosenkrantz, A. B., Labib, A., Pysarenko, K., & Prabhu, V. (2016). What do patients tweet about their mammography experience? *Academic radiology*, *23*(11), 1367-1371. | What are the themes related to patients’ experience in undergoing mammography, as expressed on Twitter? | Twitter | Content analysis of 464 tweets from July to December 2015 containing the hashtag #mammogram and relating to a patient’s mammography experience.  Strong evidence | There were  464 tweets about users’ first-hand mammography experience in the study period. | Women undergoing mammography | The most common themes were: breast compression (24.4%), advising others to undergo screening (23.9%), and the importance of the examination (18.8%). |
