# Supplementary material for: "Clicks, likes, shares and comments" a systematic review of breast cancer screening discourse in social media
Source: PLoS One. 2020 Apr 15;15(4):e0231422. doi: 10.1371/journal.pone.0231422 (PMC7159232; doi:10.1371/journal.pone.0231422)
Supplement: S2 Table — (DOCX) [file pone.0231422.s003.docx]

*2. table The volume of discourse*

| Research | Social media | Number of people posting | Number of posts/ interactions | Impression/ views |
| --- | --- | --- | --- | --- |
| Thackeray, R., Burton, S. H., Giraud-Carrier, C., Rollins, S., & Draper, C. R. (2013)  Using Twitter for breast cancer prevention: an analysis of breast cancer awareness month | Twitter  Breast Cancer Awareness Month | 797,827 | 1,351,823 | 3,028,451,603 impressions |
| Loeb et al (2017)  Tweet this: how advocacy for breast and prostate cancers stacks up on social media | Twitter  01. 01. 2012–01. 01. 2017 | 800 833 | 2 518 250 | 17 032 516 052 impressions |
| Huesch, M., Chetlen, A., Segel, J., & Schetter, S. (2017)  Frequencies of private mentions and sharing of mammography  and breast cancer terms on facebook: a pilot study | Facebook  November 15 December 15, 2016. | 1.1 million women | 1.7 million | NA |
| Basch, C. H., Hillyer, G. C., MacDonald, Z. L., & Reeves, R. (2015)  Characteristics of YouTube™ videos related to mammography | Mammography videos, YouTube | NA | 173 videos on YouTube | 23,166,120 views |
| Rosenkrantz, A. B. ,Won, E., & Doshi, A. M. (2016)  Assessing the content of YouTube videos in educating patients regarding common imaging examinations | Healthcare mammography videos YouTube | NA | 11 professional patient education videos | 151,664 views |
| Charlie, A. M., Gao, Y., & Heller, S. L. (2018)  What do patients want to know? Questions and concerns regarding mammography expressed through social media | QUORA  question and answer website  June, 2010 and February, 2017 | NA | 51 questions 172 responses | 197,620 views |
| Klippert, H., & Schaper, A. (2019).  Using Facebook to communicate mammography messages to  rural audiences | Targeted Facebook mammography education campaign as paid advertisment | An Idaho Public health Department | 5 public health breast screening messages and a questionnaire | 48,503 underscreened targeted 40+ rural Facebook users  impression: 53,317 |
| Seimenis I., Konstantinos Chouchos, K., Panos Prassopoulos, P (2018)  Radiation risk associated with X-Ray  mammography screening: Communication and  exchange of information via Tweets | mammography radiation risk related tweets  2014-2016 | 329 | 427 | NA |
| Wong et al (2016)  Sentiment analysis of breast cancer screening in the United States using Twitter | breast screening tweets | NA | 61,524 | Na |
| Lyles et al (2013)  “5 Mins of uncomfyness Is better than dealing with cancer 4 a lifetime”: an exploratory qualitative analysis of cervical and breast cancer screening dialogue on Twitter | mammography related tweets  in 5 weeks in 2012 | NA | 271 | NA |
| Nastasi et al (2017)  Breast cancer screening and social media: a content analysis  of evidence use and guideline opinions on Twitter | Twitter  5, November 2015 and 11 December 2015 | 995 | 1345 | NA |
| Rosencrantz, A. B., Anthony Labib, A., Pysarenko, K., & Prabhu, V. (2016)  What do patients tweet about their  mammography experience | Tweets about the users own screening experience |  | 464 | NA |
