# Supplementary material for: "Clicks, likes, shares and comments" a systematic review of breast cancer screening discourse in social media
Source: PLoS One. 2020 Apr 15;15(4):e0231422. doi: 10.1371/journal.pone.0231422 (PMC7159232; doi:10.1371/journal.pone.0231422)
Supplement: S3 Table — (DOCX) [file pone.0231422.s004.docx]

*3. table Participants in the discourse*

| Title | Participants in discourse (posting and viewing/ reacting to posts) |
| --- | --- |
| Huesch, M., Chetlen, A., Segel, J., & Schetter, S. (2017)  Frequencies of private mentions and sharing of mammography and breast cancer terms on Facebook: A pilot study | 1.1 million female Facebook users.  Of these, screening aged:  400 000 45-54 years’ old  300 000 55-64 years and  200 000 65+ |
| Klippert, H., & Schaper, A. (2019  Using Facebook to communicate mammography messages to rural audiences | An Idaho public health department posted 5 breast screening education posts Viewed by 48,503 over forty hard to reach, under screened rural women and men |
| Rosenkrantz, A. B. ,Won, E., & Doshi , A. M. (2016)  Assessing the content of YouTube videos in educating patients regarding common imaging examinations | The video sources for all imaging video were (11 videos):  52.4% hospitals,  19% private radiology canters  17.5% other non-profit health organizations  7.9% vendors 3.2% other for-profit health organizations |
| Basch, C. H., Hillyer, G. C., MacDonald, Z. L., & Reeves, R. (2015).  Characteristics of YouTube™ videos related to mammography | (173 videos )59% of the videos were authored by healthcare professionals. 41% were created by lay people |
| Charlie, A. M., Gao, Y., & Heller, S. L. (2018).  What do patients want to know? Questions and concerns regarding mammography expressed through social media | All 51 questions were from lay people.  172 responses.  51 responses from 19 physicians and 6 registered nurses,  121 responses from non-medical professional users. |
| Lyles, C. R., López, A., Pasick, R., & Sarkar, U. (2013).“5 mins of uncomfyness is better than dealing with cancer 4 a lifetime”: an exploratory qualitative analysis of cervical and breast cancer screening dialogue on Twitter, | (271 tweets) 73% of top tweets came from individual users, 24% from organisations and 2% from news sources. |
| Nastasi, A., Bryant, T., Canner, J. K., Dredze, M., Camp, M. S., & Nagarajan, N. (2018).Breast cancer screening and social media: A content analysis of evidence use and guideline opinions on twitter | (1345 tweets) Non-healthcare users made up 32.5% of the total,  health organizations 13.0%;  non-cancer specialist 6.5%,  cancer specialists 3% |
| Seimenis, I., Konstantinos Chouchos, K., & Panos Prassopoulos, P. (2018)  Radiation risk associated with X-Ray mammography screening: Communication and exchange of information via Tweets | (427 tweets ) 31% of all top tweets were by individuals.  24% were healthcare and news feeds.  Private companies and organizations had 19% and 15%. Specialised radiology professionals were responsible for 5% of tweets.  Non radiologist MDs posted 7% of tweets. |
| Thackeray, R., Burton, S. H., Giraud-Carrier, C., Rollins, S., & Draper, C. R. (2013).  Using Twitter for breast cancer prevention: an analysis of breast cancer awareness month | (797,827 unique users tweeted 1,351,823 breast cancer related tweets.) 93.2% of the tweeters were by individuals, 6.5 % by organizations, and 0.3% by celebrities. Organizations tweeted 10.7% of all the tweets. Celebrities 0.4% Individuals 88.9% Celebrities were 12.92 times more likely to be retweeted than organizations and 36.14 times more likely than individuals. |
| Rosencrantz A B, Anthony Labib A, Pysarenko K, Prabhu V. (2016)  *What do patients tweet about their mammography experience?* | Women undergoing mammography |
