# Supplementary material for: "Clicks, likes, shares and comments" a systematic review of breast cancer screening discourse in social media
Source: PLoS One. 2020 Apr 15;15(4):e0231422. doi: 10.1371/journal.pone.0231422 (PMC7159232; doi:10.1371/journal.pone.0231422)
Supplement: S4 Table — (DOCX) [file pone.0231422.s005.docx]

*4. table Scientific validity of discourse*

| Title | Scientific validity |
| --- | --- |
| Huesch, M., Chetlen, A., Segel, J., & Schetter, S. (2017)  Frequencies of private mentions and sharing of mammography and breast cancer terms on Facebook: A pilot study | (1.7 million interactions by more than 1.1 million female Facebook users )  6% of all top interactions in the 35-54 age group is fundamentally anti screening containing links to a natural health website |
| Rosenkrantz, A. B. ,Won, E., Doshi, A. M, (2016)  Assessing the content of YouTube videos in educating patients regarding common imaging examinations | Important issues were not addressed by a number of the videos. |
| Basch, C. H., Hillyer, G. C., MacDonald, Z. L., & Reeves, R. (2015)  Characteristics of YouTube™ videos related to mammography | There were comments that test is dangerous and even causes cancer.  The authors did not report on the scientific validity of the videos |
| Charlie, A. M., Gao, Y., & Heller, S. L. (2018)  What do patients want to know? Questions and concerns regarding mammography expressed through social media | Among lay participants, 4 of 22 (18.2%) were against screening mammography but they sited peer reviewed studies as evidence |
| Nastasi, A., Bryant, T., Canner, J. K., Dredze, M., Camp, M. S., & Nagarajan, N. (2018)  Breast cancer screening and social media: A content analysis of evidence use and guideline opinions on Twitter | (1345 mammography related tweets authored by 995 unique users)  Only 61% of non-healthcare user claim related  tweets were scientifically supported. Lay people are often sharing claims that are generally untrue .  Overall 81.6% of claims were sound scientifically  Physicians posted scientifically valid tweets (OR 11. 7)  Scientifically valid and faulty posts were just as likely to be retweeted. |
| Seimenis, I., Konstantinos Chouchos, K., Panos Prassopoulos, P .(2018)  Radiation risk associated with X-Ray mammography screening: Communication and exchange of information via Tweets | (427 tweets from 329 unique users )  23% of the posts were misleading |
