# Supplementary material for: "Clicks, likes, shares and comments" a systematic review of breast cancer screening discourse in social media
Source: PLoS One. 2020 Apr 15;15(4):e0231422. doi: 10.1371/journal.pone.0231422 (PMC7159232; doi:10.1371/journal.pone.0231422)
Supplement: S5 Table — (DOCX) [file pone.0231422.s006.docx]

5. table Main topics discussed

| Title | Main topics discussed |
| --- | --- |
| *Huesch, M., Chetlen, A., Segel, J., & Schetter, S. (2017)*  *Frequencies of private mentions and sharing of mammography and breast cancer terms on Facebook: A pilot study* | (1.7 million interactions by more than 1.1 million female Facebook users )  Of top posts:  36% e-commerce,  26% celebrity breast cancer information,  15% advocacy groups. |
| *Klippert, H., & Schaper, A. (2019)*  *Using Facebook to communicate mammography messages to rural audiences* | 6 posts of public health messages:  Mammography Importance  Mammography Purpose  Male Breast Cancer  The Women’s Health Check Link Survey link |
| *Rosenkrantz, A. B.,Won, E., & Doshi, A. M. (2016)*  *Assessing the content of YouTube videos in educating patients regarding common imaging examinations* | ( 11 Videos)Unrobing, avoiding deodorant, and possible additional images were all mentioned by 63.6% of the mammographyvideos; but dense breasts were mentioned by none of them. |
| *Basch, C. H., Hillyer, G. C., MacDonald, Z. L., Reeves, R. (2015)*  *Characteristics of YouTube™ videos related to mammography* | Of the 173 videos:  93.6% had general mammography information.  46.2% presented information about the test results  25.0% covered medical or family history  35.3% discussed pain  32.4% anxiety  29.5%. Fear 46.2 %. Results.  There were comments on safety, pain, and radiation.  Many comments were critical. |
| *Charlie, A. M., Gao, Y., & Heller, S. L. (2018)*  *What do patients want to know? Questions and concerns regarding mammography expressed through social media* | Mammographic efficacy (16 of 51 [31.4%]) and screening guidelines (10 of 51 [19.6%]) were the most common topics.  7 questions asked for help in result interpretation, 9 enquired about what to expect at screening, 4 about safety and 2 asked for advice about abnormal results |
| *The public’s response to the U.S. Preventive Services Task Force’s 2009 recommendations on mammography screening* | Screenning guidelines |
| *Lyles, C. R., López, A., Pasick, R., & Sarkar, U. (2013)*  *“5 mins of uncomfyness is better than dealing with cancer 4 a lifetime”: An exploratory qualitative analysis of cervical and breast cancer screening dialogue on Twitter,* | (271) 25% of top tweets are on personal experiences, there are some negative tweets about procedure.  promotional tweets are 18 % of the total.  Guideline critical tweets are 9%,  Jokes and sexual content is also present |
| *Nastasi, A., Bryant, T., Canner, J. K., Dredze, M., Camp, M. S., & Nagarajan, N. (2018)*  *Breast cancer screening and social media: A content analysis of evidence use and guideline opinions on Twitter* | (1345 tweets)  71.3% of the tweets had a link,  24.8% to general news sites,  (1345 tweets)  21.5% to healthcare websites,  5.2% of tweets to journal articles or guidelines.  37.9% of all tweets made testable claims,  12.9%. were on breast cancer news  11.1% were about guidelines  10.9% breast cancer personal  9.1% breast cancer other  45.3% of tweets are on BC research.  Oncologists have 6.1 times higher odds of posting about this topic followed by news organisations (3.66 OR) and physicians (OR 2.55). |
| *Seimenis, I., Konstantinos Chouchos, K., & Panos Prassopoulos, P. (2018)*  *Radiation risk associated with X-Ray mammography screening: Communication and exchange of information via Tweets* | (427 tweets from 329 unique users )  25% of tweets were remarks with no linked sources  20% had linksto peer reviewed journals,  23% had links to non- peer reviewed,  10% each were blogs, advertisements and informative websites. |
| *Thackeray, R., Burton, S. H., Giraud-Carrier, C., Rollins, S., & Draper, C. R. (2013)*  *Using Twitter for breast cancer prevention: an analysis of breast cancer awareness month* | Wearing pink, promoting breast cancer awareness walks and fundraisers were the most usual tweet topics.  Organisations and celebrities were mostly tweeting about fundraisers, early detection, and diagnoses. Individuals were tweeting about clothing and walks |
| *Rosencrantz, A. B., Anthony Labib. A., Pysarenko, K., & Prabhu, V. (2016)*  *What do patients tweet about their mammography experience?* | (464 tweets )The most common themes were: breast compression (24.4%), advising others to undergo screening (23.9%), and the importance of the examination (18.8%). |
