# Supplementary material for: "Clicks, likes, shares and comments" a systematic review of breast cancer screening discourse in social media
Source: PLoS One. 2020 Apr 15;15(4):e0231422. doi: 10.1371/journal.pone.0231422 (PMC7159232; doi:10.1371/journal.pone.0231422)
Supplement: S6 Table — (DOCX) [file pone.0231422.s007.docx]

*6. table Screening sentiment*

| Title | Screening sentiment |
| --- | --- |
| *Huesch, M., Chetlen, A., Segel, J., & Schetter, S. (2017)*  *Frequencies of private mentions and sharing of mammography and breast cancer terms on Facebook: A pilot study* | ((1.7 million interactions by more than 1.1 million female Facebook users )  Mostly positive or neutral sentiments, but 6% of all top interactions in the 35-54 age group is strongly anti screening |
| *Klippert, H., & Schaper, A. (2019)*  *Using Facebook to communicate mammography messages to rural audiences* | The 1,189 reactions to the posts were mostly positive |
| *Basch, C. H., Hillyer, G. C., MacDonald, Z. L., & Reeves, R. (2015)*  *Characteristics of YouTube™ videos related to mammography* | (173 videos ) ‘thumbs up’ outweigh ‘thumbs down’ 15:1  Some comments say mammography is dangerous and causes cancer |
| *Charlie, A. M., Gao, Y., & Heller, S. L. (2018)*  *What do patients want to Know? Questions and concerns regarding mammography expressed through social media* | Although critical of guidelines, the majority of participants were supportive of mammography, with the exception of 4 of the 22 lay participants. (18.2%) |
| *Wong, K. O., Davis, F. G., Zaïane, O. R., Yasui, Y., Dietz, J., Aveiro, D., ... & Bernardino, J. (2016). Sentiment analysis of breast cancer screening in the United States using Twitter* | There were 29,034 neutral, 21,561 positive and 4,069 negative tweets*.* |
| *Squiers, L. B., Holden, D. J., Dolina, S. E., Kim, A. E., Bann, C. M., & Renaud, J. M. (2011)*  *The public’s response to the U.S. Preventive Services Task Force’s 2009 recommendations on mammography screening* | (82 tweets and 71 blog posts)  48.8% of the tweets were neutral, 36.6% unsupportive of the changed guideline and just 2.4% supportive, while 12.2% were confused  Blogs: 17%  say postponing the recommended age of screening leads to breast cancer deaths.  31% say changes are due to government rationing of resources. 14% changes had a scientific rationale.  66.2% of blog post are negative |
| *Lyles, C. R., López, A., Pasick, R., & Sarkar, U. (2013)*  *“5 Mins of uncomfyness is better than dealing with cancer 4 a lifetime”: an exploratory qualitative analysis of cervical and breast cancer screening dialogue on Twitter,* | (271 ) Guideline critical tweets are 9% of the tweets  25% of tweets are about personal experience. About 10% of these tweets mention fear, pain and anxiety. |
| *Nastasi, A., Bryant, T., Canner, J. K., Dredze, M., Camp, M. S., & Nagarajan, N. (2018)*  *Breast cancer screening and social media: A content analysis of evidence use and guideline opinions on Twitter* | (1345) Emotion associated with tweet in general:  Neutral 95%  Negative 3.2%  Positive 1.7%  Out of 137 guideline related tweets, 4 were approving of the new  guidelines, 39 were disapproving, and 70 were neutral.   20 voiced confusions over guidelines.  39% of tweets by non-healthcare users were not backed by the scientific community. |
| *Seimenis, I., Konstantinos Chouchos, K., & Panos Prassopoulos, P. (2018)*  *Radiation risk associated with X-Ray mammography screening: Communication and exchange of information via Tweets* | (427 tweets from 329 unique users )  42% of the tweets were favourable to mammography, 32% neutral and 26% were negative..  62% of unfavourable post were  from the general public while only 17% of the favourable posts were by them.   6 physicians  had unfavourable comments on mammography. 73% of total tweets were informative,  23% were misleading and 4% were questions |
| *Thackeray, R., Burton, S. H., Giraud-Carrier, C., Rollins, S., & Draper, C. R. (2013)*  *Using Twitter for breast cancer prevention: an analysis of breast cancer awareness month* | Tweets are mostly positive |
| *Rosencrantz, A. B., Anthony Labib, A., Pysarenko, K., & Prabhu, V. (2016)*  *What do patients tweet about their mammography experience?* | Tweets are mosty positive |
